# {μ-*N*-[(Diphenyl­phosphino)meth­yl]pyridin-2-amine-κ^2^ *N* ^1^:*P*}bis­{[2-(2,2′-bipyridin-6-yl)phenyl-κ^3^ *N*,*N*′,*C* ^1^]platinum(II)} bis­(perchlorate)

**DOI:** 10.1107/S1600536808024525

**Published:** 2008-08-09

**Authors:** Xiang-Dang Du, Juan Mo, Xin-Sheng Li, Yu-Shan Pan, Su-Mei Zhang

**Affiliations:** a College of Animal Husbandry and Veterinary Studies, Henan Agricultural University, Zhengzhou, Henan 450002, People’s Republic of China

## Abstract

The title compound, [Pt_2_(C_16_H_11_N_2_)_2_(C_18_H_17_N_2_P)](ClO_4_)_2_, contains two Pt^II^ atoms, bridged by an *N*-[(diphenyl­phosphino)meth­yl]pyridin-2-amine (dppmp) ligand. One Pt atom is coordinated by one P atom from the dppmp ligand, and one C atom and two N atoms from a 6-phenyl-2,2′-bipyridine (pbpy) ligand in a square-planar geometry. The other Pt atom is coordinated by one N atom from the dppmp ligand, and one C atom and two N atoms from another pbpy ligand in a square-planar geometry. There are intra­molecular π–π inter­actions between the pbpy ligands, with a centroid–centroid distance of 3.62 (1) Å between two pyridyl rings. The oxygen atoms of both perchlorate anions are disordered, each over two different positions [occupanicies 0.49 (3)/0.51 (3) and 0.48 (2)/0.52 (2)].

## Related literature

For related literature, see: Braunstein *et al.* (1997 ▶); Catalano *et al.* (2001 ▶); Durran *et al.* (2000 ▶); Field *et al.* (1997 ▶); Kuang *et al.* (1998 ▶); Li *et al.* (1996 ▶); Newkome (1993 ▶).
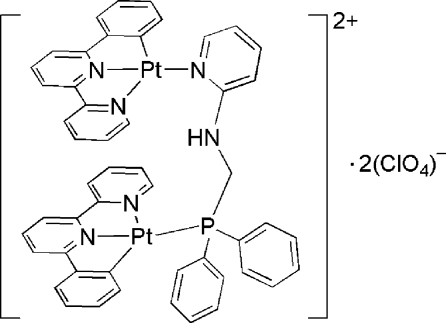


## Experimental

### 

#### Crystal data

[Pt_2_(C_16_H_11_N_2_)_2_(C_18_H_17_N_2_P)](ClO_4_)_2_
*M* *_r_* = 1343.92
Monoclinic, 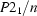

*a* = 14.845 (3) Å
*b* = 17.927 (4) Å
*c* = 18.481 (4) Å
β = 109.697 (3)°
*V* = 4630.5 (18) Å^3^
*Z* = 4
Mo *K*α radiation
μ = 6.25 mm^−1^
*T* = 294 (2) K
0.40 × 0.20 × 0.10 mm

#### Data collection

Bruker SMART APEX CCD area-detector diffractometer
Absorption correction: multi-scan (*SADABS*; Sheldrick, 1996 ▶) *T*_min_ = 0.225, *T*_max_ = 0.533
22850 measured reflections
8097 independent reflections
5253 reflections with *I* > 2σ(*I*)
*R*_int_ = 0.090

#### Refinement

*R*[*F* ^2^ > 2σ(*F* ^2^)] = 0.075
*wR*(*F* ^2^) = 0.219
*S* = 1.03
8097 reflections
696 parameters
16 restraints
H-atom parameters constrained
Δρ_max_ = 2.85 e Å^−3^
Δρ_min_ = −2.38 e Å^−3^

### 

Data collection: *SMART* (Bruker, 2007 ▶); cell refinement: *SAINT* (Bruker, 2007 ▶); data reduction: *SAINT*; program(s) used to solve structure: *SHELXS97* (Sheldrick, 2008 ▶); program(s) used to refine structure: *SHELXL97* (Sheldrick, 2008 ▶); molecular graphics: *SHELXTL* (Sheldrick, 2008 ▶); software used to prepare material for publication: *SHELXTL*.

## Supplementary Material

Crystal structure: contains datablocks global, I. DOI: 10.1107/S1600536808024525/hy2138sup1.cif

Structure factors: contains datablocks I. DOI: 10.1107/S1600536808024525/hy2138Isup2.hkl

Additional supplementary materials:  crystallographic information; 3D view; checkCIF report

## Figures and Tables

**Table:** 

Pt1—N2	1.928 (13)
Pt1—C16	1.990 (15)
Pt1—N3	2.038 (14)
Pt1—N1	2.079 (11)
Pt2—N6	1.993 (13)
Pt2—C50	2.014 (14)
Pt2—N5	2.164 (14)
Pt2—P1	2.241 (4)

**Table:** 

N2—Pt1—C16	82.8 (6)
N2—Pt1—N3	178.1 (4)
C16—Pt1—N3	98.7 (6)
N2—Pt1—N1	79.5 (5)
C16—Pt1—N1	162.3 (6)
N3—Pt1—N1	99.0 (5)
N6—Pt2—C50	81.7 (6)
N6—Pt2—N5	77.4 (5)
C50—Pt2—N5	158.8 (6)
N6—Pt2—P1	173.4 (4)
C50—Pt2—P1	95.0 (5)
N5—Pt2—P1	106.2 (4)

**Fig. 1.**
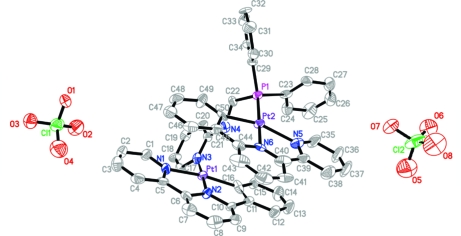
Molecular structure of the title compound. Displacement ellipsoids are drawn at the 40% probability level. H atoms and the minor disordered positions of the perchlorate anions have been omitted for clarity.

## supplementary crystallographic information

### Comment

Pyridylphosphines have induced much interest as excellent ligands with both P– and N-donor centres (Kuang *et al.*, 1998; Newkome, 1993). These pyridylphosphines display various coordination modes: P-coordination, N-coordination, P,*N*-chelating and P,*N*-bridging (Braunstein *et al.*, 1997; Catalano *et al.*, 2001; Durran *et al.*, 2000; Field *et al.*, 1997; Li *et al.*, 1996). Here, we report the crystal structure of the title compound, in which two Pt^II^ atoms are bridged by an *N*-[(diphenylphosphino)methyl]pyridin-2-amine (dppmp) ligand.

In the title compound (Fig. 1), the Pt1 atom is coordinated by one pyridyl N atom from the dppmp ligand, and one C atom and two N atoms from a 6-phenyl-2,2'-bipyridine (pbpy) ligand in a square-planar geometry (Table 1). The Pt2 atom is coordinated by one P atom from the dppmp ligand, and one C atom and two N atoms from another pbpy ligand in a square-planar geometry. The atoms Pt1, N1, N2, N3 and C16 show a mean deviation of 0.01 (1)Å from the least-squares plane through them. The planarity of the coordination geometry around the Pt2 atom was accessed by fitting a least-squares plane to the atoms Pt2, P1, N5, N6 and C50, which show a mean deviation of 0.08 (1) Å. Dihedral angle between the planes defined by two Pt atoms and the corresponding coordinated atoms is 16.6 (2)°.

### Experimental

A solution of K_2_(PtCl_4_) (0.088 g, 0.212 mmol) in water (5 ml) was treated with a solution of pbpy (0.052 g, 0.224 mmol) in MeCN (5 ml) and the mixture was heated to reflux for 20 h, allowing MeCN to evaporate slowly. The orange solid so obtained was collected by filtration, washed with water and water–EtOH (5:l, *v*/*v*) and dried *in vacuo*, yielding Pt(pbpy)Cl (yield 91%, 0.089 g). A 15 ml MeCN solution of Pt(pbpy)Cl (0.046 g, 0.1 mmol) was added to a 20 ml MeCN solution of dppmp (0.015 g, 0.05 mmol). The mixture was stirred for 10 h and then LiClO_4_ (0.021 g, 0.2 mmol) was added. Yellow crystals suitable for X-ray diffraction were formed by vapour diffusion of diethyl ether into the MeCN solution.

### Refinement

H atoms were positioned geometrically and refined as riding atoms, with C—H = 0.93 (aromatic) and 0.97 (CH_2_) Å, and N—H = 0.86 Å, and with *U*_iso_(H) = 1.2*U*_eq_(C,*N*). The highest residual electron density was found 0.98 Å from atom Pt2 and the deepest hole 1.00 Å from atom Pt2. Each perchlorate anion is disordered over two different orientations. The Cl—O distances were restrained to 1.44 (3) Å.

### Crystal data

**Table:**

[Pt_2_(C_16_H_11_N_2_)_2_(C_18_H_17_N_2_P)](ClO_4_)_2_	*F*_000_ = 2600
*M**_r_* = 1343.92	*D*_x_ = 1.928 Mg m^−^^3^
Monoclinic, *P*2_1_/*n*	Mo *K*α radiation λ = 0.71073 Å
Hall symbol: -P 2yn	Cell parameters from 7716 reflections
*a* = 14.845 (3) Å	θ = 2.3–26.6º
*b* = 17.927 (4) Å	µ = 6.25 mm^−^^1^
*c* = 18.481 (4) Å	*T* = 294 (2) K
β = 109.697 (3)º	Block, yellow
*V* = 4630.5 (18) Å^3^	0.40 × 0.20 × 0.10 mm
*Z* = 4	

### Data collection

**Table:**

Bruker SMART APEX CCD area-detector diffractometer	8097 independent reflections
Radiation source: fine-focus sealed tube	5253 reflections with *I* > 2σ(*I*)
Monochromator: graphite	*R*_int_ = 0.090
*T* = 294(2) K	θ_max_ = 25.0º
φ and ω scans	θ_min_ = 1.6º
Absorption correction: multi-scan(SADABS; Sheldrick, 1996)	*h* = −17→16
*T*_min_ = 0.225, *T*_max_ = 0.534	*k* = −17→21
22850 measured reflections	*l* = −20→21

### Refinement

**Table:**

Refinement on *F*^2^	Secondary atom site location: difference Fourier map
Least-squares matrix: full	Hydrogen site location: inferred from neighbouring sites
*R*[*F*^2^ > 2σ(*F*^2^)] = 0.075	H-atom parameters constrained
*wR*(*F*^2^) = 0.219	*w* = 1/[σ^2^(*F*_o_^2^) + (0.0656*P*)^2^ + 39.0816*P*] where *P* = (*F*_o_^2^ + 2*F*_c_^2^)/3
*S* = 1.03	(Δ/σ)_max_ = 0.001
8097 reflections	Δρ_max_ = 2.85 e Å^−^^3^
696 parameters	Δρ_min_ = −2.38 e Å^−^^3^
16 restraints	Extinction correction: none
Primary atom site location: structure-invariant direct methods	

### Fractional atomic coordinates and isotropic or equivalent isotropic displacement parameters (Å^2^)

**Table:**

	*x*	*y*	*z*	*U*_iso_*/*U*_eq_	Occ. (<1)
Pt1	0.96899 (4)	0.15372 (3)	0.47609 (3)	0.0324 (2)	
Pt2	0.71657 (4)	0.21672 (3)	0.51620 (3)	0.0326 (2)	
P1	0.7571 (3)	0.3339 (2)	0.55627 (19)	0.0325 (8)	
N1	0.9510 (9)	0.1645 (8)	0.3600 (6)	0.040 (3)	
N2	0.8943 (8)	0.0657 (8)	0.4355 (6)	0.033 (3)	
N3	1.0503 (9)	0.2462 (8)	0.5165 (5)	0.038 (3)	
N4	0.9260 (9)	0.3020 (8)	0.5450 (7)	0.040 (3)	
H4	0.9029	0.2582	0.5464	0.048*	
N5	0.7113 (10)	0.1553 (8)	0.6155 (8)	0.047 (3)	
N6	0.6669 (8)	0.1175 (7)	0.4717 (7)	0.033 (3)	
C1	0.9763 (13)	0.2199 (10)	0.3226 (9)	0.047 (4)	
H1	1.0069	0.2613	0.3504	0.056*	
C2	0.9588 (14)	0.2184 (12)	0.2437 (10)	0.060 (5)	
H2	0.9781	0.2579	0.2198	0.072*	
C3	0.9137 (15)	0.1595 (13)	0.2027 (9)	0.065 (6)	
H3	0.9037	0.1572	0.1503	0.078*	
C4	0.8816 (12)	0.1013 (11)	0.2385 (9)	0.053 (5)	
H4A	0.8477	0.0616	0.2095	0.064*	
C5	0.9009 (11)	0.1032 (9)	0.3175 (8)	0.040 (4)	
C6	0.8704 (10)	0.0466 (10)	0.3612 (7)	0.039 (4)	
C7	0.8170 (11)	−0.0157 (11)	0.3327 (9)	0.053 (5)	
H7	0.7987	−0.0268	0.2806	0.063*	
C8	0.7905 (12)	−0.0622 (11)	0.3821 (10)	0.056 (5)	
H8	0.7547	−0.1050	0.3638	0.068*	
C9	0.8179 (10)	−0.0437 (10)	0.4582 (9)	0.042 (4)	
H9	0.7979	−0.0733	0.4912	0.050*	
C10	0.8741 (10)	0.0176 (9)	0.4873 (7)	0.034 (3)	
C11	0.9076 (10)	0.0489 (9)	0.5636 (8)	0.035 (3)	
C12	0.8939 (11)	0.0107 (11)	0.6275 (8)	0.047 (4)	
H12	0.8600	−0.0338	0.6203	0.056*	
C13	0.9321 (14)	0.0416 (13)	0.6992 (9)	0.063 (5)	
H13	0.9226	0.0182	0.7409	0.076*	
C14	0.9833 (13)	0.1055 (13)	0.7101 (9)	0.061 (5)	
H14	1.0103	0.1242	0.7597	0.073*	
C15	0.9969 (12)	0.1443 (10)	0.6490 (8)	0.045 (4)	
H15	1.0298	0.1894	0.6580	0.054*	
C16	0.9616 (9)	0.1158 (11)	0.5751 (7)	0.042 (4)	
C17	1.1411 (10)	0.2502 (11)	0.5117 (9)	0.044 (4)	
H17	1.1649	0.2090	0.4933	0.053*	
C18	1.1961 (13)	0.3111 (10)	0.5326 (10)	0.049 (4)	
H18	1.2581	0.3103	0.5311	0.059*	
C19	1.1611 (10)	0.3767 (10)	0.5568 (8)	0.041 (4)	
H19	1.1970	0.4203	0.5681	0.049*	
C20	1.0709 (11)	0.3726 (9)	0.5628 (7)	0.039 (4)	
H20	1.0464	0.4131	0.5814	0.047*	
C21	1.0161 (10)	0.3071 (9)	0.5406 (6)	0.030 (3)	
C22	0.8681 (9)	0.3651 (9)	0.5475 (8)	0.034 (3)	
H22A	0.8554	0.3943	0.5009	0.041*	
H22B	0.9018	0.3965	0.5909	0.041*	
C23	0.7700 (12)	0.3500 (8)	0.6562 (8)	0.040 (4)	
C24	0.8588 (12)	0.3455 (11)	0.7132 (9)	0.054 (5)	
H24	0.9141	0.3432	0.7003	0.065*	
C25	0.8646 (16)	0.3446 (13)	0.7892 (10)	0.077 (7)	
H25	0.9242	0.3390	0.8271	0.092*	
C26	0.7854 (14)	0.3515 (12)	0.8103 (10)	0.059 (5)	
H26	0.7902	0.3514	0.8618	0.070*	
C27	0.6987 (15)	0.3588 (12)	0.7532 (9)	0.064 (6)	
H27	0.6440	0.3635	0.7666	0.077*	
C28	0.6894 (13)	0.3592 (12)	0.6757 (9)	0.055 (5)	
H28	0.6298	0.3656	0.6380	0.067*	
C29	0.6740 (10)	0.4050 (9)	0.5055 (7)	0.035 (3)	
C30	0.5850 (11)	0.3829 (11)	0.4536 (7)	0.043 (4)	
H30	0.5689	0.3328	0.4450	0.051*	
C31	0.5226 (16)	0.4377 (15)	0.4159 (10)	0.074 (7)	
H31	0.4637	0.4239	0.3807	0.089*	
C32	0.5421 (17)	0.5077 (13)	0.4274 (12)	0.072 (7)	
H32	0.4966	0.5432	0.4022	0.086*	
C33	0.6317 (15)	0.5307 (12)	0.4777 (13)	0.069 (6)	
H33	0.6462	0.5813	0.4842	0.083*	
C34	0.6956 (13)	0.4806 (10)	0.5160 (10)	0.048 (4)	
H34	0.7547	0.4959	0.5498	0.058*	
C35	0.7382 (16)	0.1770 (13)	0.6886 (10)	0.067 (6)	
H35	0.7650	0.2241	0.7019	0.080*	
C36	0.7269 (18)	0.1308 (15)	0.7452 (13)	0.081 (7)	
H36	0.7504	0.1458	0.7963	0.097*	
C37	0.6828 (16)	0.0650 (16)	0.7270 (13)	0.085 (8)	
H37	0.6692	0.0361	0.7638	0.102*	
C38	0.6578 (15)	0.0413 (13)	0.6510 (12)	0.070 (6)	
H38	0.6328	−0.0062	0.6375	0.084*	
C39	0.6702 (11)	0.0888 (9)	0.5949 (9)	0.044 (4)	
C40	0.6436 (12)	0.0660 (11)	0.5145 (11)	0.055 (5)	
C41	0.6049 (13)	−0.0013 (11)	0.4835 (11)	0.059 (5)	
H41	0.5944	−0.0383	0.5151	0.070*	
C42	0.5824 (13)	−0.0137 (12)	0.4087 (13)	0.065 (6)	
H42	0.5563	−0.0591	0.3875	0.078*	
C43	0.5988 (13)	0.0432 (12)	0.3620 (12)	0.066 (6)	
H43	0.5805	0.0366	0.3091	0.079*	
C44	0.6428 (10)	0.1095 (10)	0.3956 (9)	0.042 (4)	
C45	0.6726 (11)	0.1717 (10)	0.3581 (8)	0.038 (4)	
C46	0.6645 (13)	0.1717 (12)	0.2834 (9)	0.056 (5)	
H46	0.6359	0.1316	0.2523	0.068*	
C47	0.6988 (14)	0.2316 (14)	0.2526 (9)	0.067 (6)	
H47	0.6976	0.2300	0.2019	0.081*	
C48	0.7345 (13)	0.2930 (12)	0.2973 (9)	0.060 (5)	
H48	0.7558	0.3339	0.2765	0.072*	
C49	0.7389 (12)	0.2942 (11)	0.3716 (8)	0.049 (4)	
H49	0.7612	0.3374	0.3999	0.059*	
C50	0.7124 (10)	0.2355 (9)	0.4077 (7)	0.034 (4)	
Cl1	0.9289 (4)	0.2821 (3)	0.0174 (3)	0.0685 (14)	
Cl2	0.4595 (5)	0.0612 (3)	0.8325 (2)	0.0868 (19)	
O1	0.8360 (8)	0.3152 (11)	0.0026 (12)	0.045 (7)	0.49 (3)
O2	0.9880 (13)	0.3037 (16)	0.0961 (7)	0.085 (11)	0.49 (3)
O3	0.9732 (14)	0.3115 (14)	−0.0339 (11)	0.066 (10)	0.49 (3)
O4	0.9225 (17)	0.2030 (5)	0.0144 (18)	0.099 (12)	0.49 (3)
O1'	0.8580 (14)	0.3177 (12)	0.0424 (16)	0.084 (10)	0.51 (3)
O2'	0.9950 (14)	0.2415 (15)	0.0795 (12)	0.115 (12)	0.51 (3)
O3'	0.9770 (13)	0.3359 (10)	−0.0133 (11)	0.043 (6)	0.51 (3)
O4'	0.8809 (18)	0.2289 (13)	−0.0433 (12)	0.105 (12)	0.51 (3)
O5	0.5418 (15)	0.0136 (14)	0.8519 (16)	0.126 (14)	0.52 (2)
O6	0.465 (2)	0.1108 (12)	0.8945 (10)	0.089 (10)	0.52 (2)
O7	0.452 (2)	0.1028 (12)	0.7643 (9)	0.070 (9)	0.52 (2)
O8	0.3747 (15)	0.0152 (14)	0.8177 (17)	0.150 (17)	0.52 (2)
O5'	0.4931 (16)	−0.0120 (7)	0.8234 (11)	0.058 (8)	0.48 (2)
O6'	0.5415 (14)	0.1089 (12)	0.8684 (15)	0.125 (15)	0.48 (2)
O7'	0.4045 (15)	0.0923 (11)	0.7596 (8)	0.052 (7)	0.48 (2)
O8'	0.4025 (19)	0.0581 (17)	0.8818 (14)	0.163 (18)	0.48 (2)

### Atomic displacement parameters (Å^2^)

**Table:**

	*U*^11^	*U*^22^	*U*^33^	*U*^12^	*U*^13^	*U*^23^
Pt1	0.0415 (4)	0.0279 (4)	0.0241 (3)	0.0019 (2)	0.0060 (2)	−0.0019 (2)
Pt2	0.0420 (4)	0.0225 (4)	0.0301 (3)	−0.0007 (2)	0.0079 (2)	−0.0017 (2)
P1	0.044 (2)	0.025 (2)	0.0253 (17)	0.0033 (17)	0.0066 (14)	−0.0016 (14)
N1	0.057 (8)	0.037 (9)	0.030 (6)	−0.001 (7)	0.021 (6)	−0.006 (5)
N2	0.035 (7)	0.039 (8)	0.020 (5)	−0.002 (6)	0.003 (4)	0.002 (5)
N3	0.048 (7)	0.049 (9)	0.013 (5)	0.001 (7)	0.004 (5)	−0.001 (5)
N4	0.046 (8)	0.034 (8)	0.044 (7)	−0.011 (6)	0.021 (6)	−0.007 (6)
N5	0.046 (8)	0.033 (9)	0.059 (9)	0.000 (6)	0.012 (6)	0.006 (6)
N6	0.030 (7)	0.026 (7)	0.044 (7)	0.009 (5)	0.014 (5)	−0.007 (5)
C1	0.066 (11)	0.035 (11)	0.040 (8)	−0.002 (9)	0.019 (7)	0.003 (7)
C2	0.078 (13)	0.064 (16)	0.047 (10)	0.018 (11)	0.032 (9)	0.021 (9)
C3	0.087 (14)	0.078 (17)	0.026 (8)	0.003 (12)	0.014 (8)	−0.005 (9)
C4	0.066 (11)	0.042 (12)	0.041 (9)	0.015 (9)	0.003 (7)	−0.006 (8)
C5	0.052 (9)	0.029 (9)	0.034 (7)	0.017 (8)	0.009 (6)	−0.002 (6)
C6	0.040 (8)	0.047 (11)	0.027 (7)	0.018 (8)	0.006 (6)	−0.006 (6)
C7	0.050 (10)	0.055 (13)	0.041 (9)	0.004 (9)	−0.001 (7)	−0.016 (8)
C8	0.060 (11)	0.037 (11)	0.059 (11)	−0.012 (9)	0.004 (8)	−0.019 (8)
C9	0.037 (8)	0.033 (10)	0.063 (10)	−0.002 (7)	0.025 (7)	0.005 (7)
C10	0.044 (8)	0.023 (8)	0.034 (7)	0.003 (7)	0.011 (6)	0.006 (6)
C11	0.035 (8)	0.033 (9)	0.035 (7)	0.005 (7)	0.008 (6)	0.006 (6)
C12	0.043 (9)	0.054 (12)	0.041 (8)	0.001 (8)	0.010 (7)	0.013 (8)
C13	0.082 (13)	0.074 (16)	0.034 (9)	0.002 (12)	0.020 (8)	0.015 (9)
C14	0.075 (13)	0.078 (17)	0.028 (8)	−0.007 (12)	0.013 (7)	−0.003 (8)
C15	0.061 (10)	0.043 (11)	0.026 (7)	−0.002 (8)	0.009 (7)	0.003 (6)
C16	0.017 (7)	0.079 (14)	0.026 (7)	0.000 (8)	0.002 (5)	−0.015 (7)
C17	0.035 (9)	0.052 (12)	0.059 (9)	0.011 (8)	0.033 (7)	0.011 (8)
C18	0.055 (10)	0.027 (10)	0.063 (10)	−0.002 (8)	0.015 (8)	0.004 (7)
C19	0.031 (8)	0.043 (11)	0.043 (8)	−0.020 (7)	0.006 (6)	−0.001 (7)
C20	0.059 (10)	0.023 (9)	0.027 (7)	−0.013 (7)	0.002 (6)	−0.004 (6)
C21	0.039 (8)	0.034 (9)	0.015 (6)	0.001 (7)	0.005 (5)	−0.010 (5)
C22	0.031 (8)	0.046 (10)	0.028 (7)	0.004 (7)	0.013 (6)	−0.002 (6)
C23	0.069 (11)	0.012 (8)	0.033 (7)	0.003 (7)	0.008 (7)	−0.010 (5)
C24	0.050 (10)	0.068 (15)	0.039 (9)	−0.005 (9)	0.009 (7)	0.000 (8)
C25	0.095 (16)	0.085 (19)	0.029 (9)	−0.025 (13)	−0.007 (9)	−0.012 (9)
C26	0.079 (14)	0.062 (15)	0.037 (9)	−0.010 (11)	0.022 (9)	−0.016 (8)
C27	0.094 (15)	0.067 (16)	0.037 (9)	0.004 (11)	0.031 (9)	−0.007 (8)
C28	0.062 (11)	0.064 (14)	0.040 (9)	0.031 (10)	0.017 (8)	0.011 (8)
C29	0.041 (8)	0.033 (10)	0.028 (7)	0.016 (7)	0.009 (6)	0.003 (6)
C30	0.053 (10)	0.051 (12)	0.021 (7)	0.013 (8)	0.008 (6)	−0.004 (6)
C31	0.092 (15)	0.09 (2)	0.035 (9)	0.043 (14)	0.015 (9)	0.016 (10)
C32	0.098 (17)	0.050 (15)	0.083 (14)	0.053 (13)	0.050 (13)	0.046 (11)
C33	0.084 (15)	0.048 (15)	0.096 (15)	0.008 (12)	0.058 (13)	0.039 (12)
C34	0.066 (11)	0.031 (11)	0.063 (10)	0.018 (8)	0.042 (9)	0.016 (8)
C35	0.111 (16)	0.051 (13)	0.036 (9)	0.010 (12)	0.021 (9)	0.015 (8)
C36	0.123 (19)	0.067 (17)	0.065 (13)	−0.010 (15)	0.048 (13)	0.026 (11)
C37	0.094 (16)	0.09 (2)	0.083 (16)	0.003 (14)	0.044 (13)	0.055 (14)
C38	0.092 (15)	0.041 (13)	0.081 (14)	−0.008 (11)	0.034 (11)	0.006 (10)
C39	0.046 (9)	0.024 (9)	0.063 (10)	0.002 (7)	0.020 (7)	0.000 (7)
C40	0.047 (10)	0.033 (11)	0.070 (11)	−0.004 (8)	0.001 (8)	−0.015 (9)
C41	0.068 (12)	0.028 (11)	0.075 (12)	0.014 (9)	0.018 (9)	−0.003 (9)
C42	0.049 (11)	0.039 (13)	0.106 (16)	−0.006 (9)	0.022 (10)	−0.020 (11)
C43	0.058 (11)	0.044 (13)	0.074 (12)	−0.003 (10)	−0.007 (9)	−0.037 (10)
C44	0.027 (8)	0.044 (11)	0.048 (9)	0.007 (7)	0.005 (6)	−0.009 (7)
C45	0.041 (9)	0.036 (10)	0.026 (7)	0.007 (7)	−0.002 (6)	−0.003 (6)
C46	0.069 (12)	0.051 (13)	0.029 (8)	0.026 (10)	−0.010 (7)	−0.013 (8)
C47	0.084 (14)	0.083 (18)	0.026 (8)	0.039 (13)	0.007 (8)	0.007 (9)
C48	0.071 (12)	0.053 (13)	0.043 (9)	0.020 (10)	0.004 (8)	0.006 (8)
C49	0.065 (11)	0.050 (12)	0.026 (7)	−0.016 (9)	0.006 (7)	0.001 (7)
C50	0.034 (8)	0.040 (10)	0.030 (7)	0.008 (7)	0.012 (6)	−0.020 (6)
Cl1	0.070 (3)	0.057 (4)	0.091 (4)	0.007 (3)	0.044 (3)	0.023 (3)
Cl2	0.158 (6)	0.043 (3)	0.038 (2)	0.008 (3)	0.005 (3)	0.000 (2)
O1	0.043 (10)	0.049 (11)	0.046 (10)	−0.003 (8)	0.021 (7)	−0.006 (7)
O2	0.092 (13)	0.083 (14)	0.076 (13)	0.002 (9)	0.022 (9)	0.009 (9)
O3	0.071 (12)	0.066 (13)	0.059 (12)	−0.008 (9)	0.021 (8)	−0.007 (9)
O4	0.099 (14)	0.097 (15)	0.110 (15)	0.004 (9)	0.046 (10)	0.002 (9)
O1'	0.080 (13)	0.087 (14)	0.088 (13)	−0.008 (9)	0.033 (9)	−0.001 (9)
O2'	0.119 (14)	0.116 (15)	0.112 (14)	0.010 (9)	0.041 (10)	0.016 (9)
O3'	0.043 (9)	0.042 (10)	0.043 (9)	−0.010 (7)	0.014 (7)	−0.008 (8)
O4'	0.104 (14)	0.105 (16)	0.107 (15)	−0.004 (9)	0.034 (10)	−0.001 (9)
O5	0.123 (16)	0.120 (17)	0.129 (16)	0.006 (10)	0.034 (10)	0.009 (10)
O6	0.100 (13)	0.085 (13)	0.073 (12)	−0.003 (9)	0.018 (8)	−0.013 (9)
O7	0.081 (12)	0.055 (12)	0.067 (11)	−0.005 (9)	0.018 (8)	0.012 (8)
O8	0.153 (18)	0.143 (19)	0.144 (19)	−0.007 (10)	0.038 (10)	0.002 (10)
O5'	0.069 (11)	0.046 (11)	0.047 (10)	0.006 (8)	0.003 (7)	−0.001 (8)
O6'	0.129 (17)	0.118 (17)	0.120 (17)	−0.004 (10)	0.030 (10)	−0.006 (10)
O7'	0.043 (10)	0.047 (11)	0.057 (10)	−0.002 (8)	0.007 (7)	0.006 (8)
O8'	0.167 (19)	0.17 (2)	0.161 (19)	0.005 (10)	0.062 (11)	0.007 (10)

### Geometric parameters (Å, °)

**Table:**

Pt1—N2	1.928 (13)	C24—C25	1.38 (2)
Pt1—C16	1.990 (15)	C24—H24	0.9300
Pt1—N3	2.038 (14)	C25—C26	1.36 (3)
Pt1—N1	2.079 (11)	C25—H25	0.9300
Pt2—N6	1.993 (13)	C26—C27	1.37 (3)
Pt2—C50	2.014 (14)	C26—H26	0.9300
Pt2—N5	2.164 (14)	C27—C28	1.39 (2)
Pt2—P1	2.241 (4)	C27—H27	0.9300
P1—C22	1.799 (14)	C28—H28	0.9300
P1—C29	1.802 (14)	C29—C34	1.39 (2)
P1—C23	1.814 (14)	C29—C30	1.40 (2)
N1—C1	1.34 (2)	C30—C31	1.37 (3)
N1—C5	1.41 (2)	C30—H30	0.9300
N2—C6	1.342 (17)	C31—C32	1.29 (3)
N2—C10	1.394 (18)	C31—H31	0.9300
N3—C21	1.342 (19)	C32—C33	1.40 (3)
N3—C17	1.381 (19)	C32—H32	0.9300
N4—C21	1.370 (18)	C33—C34	1.33 (2)
N4—C22	1.43 (2)	C33—H33	0.9300
N4—H4	0.8600	C34—H34	0.9300
N5—C35	1.33 (2)	C35—C36	1.39 (3)
N5—C39	1.34 (2)	C35—H35	0.9300
N6—C40	1.33 (2)	C36—C37	1.34 (4)
N6—C44	1.338 (19)	C36—H36	0.9300
C1—C2	1.39 (2)	C37—C38	1.39 (3)
C1—H1	0.9300	C37—H37	0.9300
C2—C3	1.34 (3)	C38—C39	1.40 (3)
C2—H2	0.9300	C38—H38	0.9300
C3—C4	1.40 (3)	C39—C40	1.46 (2)
C3—H3	0.9300	C40—C41	1.38 (3)
C4—C5	1.39 (2)	C41—C42	1.33 (3)
C4—H4A	0.9300	C41—H41	0.9300
C5—C6	1.46 (2)	C42—C43	1.41 (3)
C6—C7	1.37 (2)	C42—H42	0.9300
C7—C8	1.39 (3)	C43—C44	1.40 (2)
C7—H7	0.9300	C43—H43	0.9300
C8—C9	1.37 (2)	C44—C45	1.46 (2)
C8—H8	0.9300	C45—C46	1.35 (2)
C9—C10	1.37 (2)	C45—C50	1.46 (2)
C9—H9	0.9300	C46—C47	1.39 (3)
C10—C11	1.44 (2)	C46—H46	0.9300
C11—C16	1.42 (2)	C47—C48	1.37 (3)
C11—C12	1.44 (2)	C47—H47	0.9300
C12—C13	1.37 (2)	C48—C49	1.35 (2)
C12—H12	0.9300	C48—H48	0.9300
C13—C14	1.35 (3)	C49—C50	1.37 (2)
C13—H13	0.9300	C49—H49	0.9300
C14—C15	1.40 (2)	Cl1—O4	1.421 (8)
C14—H14	0.9300	Cl1—O3	1.424 (8)
C15—C16	1.39 (2)	Cl1—O3'	1.427 (8)
C15—H15	0.9300	Cl1—O2'	1.432 (8)
C17—C18	1.34 (2)	Cl1—O1'	1.434 (8)
C17—H17	0.9300	Cl1—O1	1.440 (8)
C18—C19	1.42 (3)	Cl1—O4'	1.461 (9)
C18—H18	0.9300	Cl1—O2	1.474 (8)
C19—C20	1.38 (2)	Cl2—O6	1.430 (9)
C19—H19	0.9300	Cl2—O7'	1.431 (8)
C20—C21	1.41 (2)	Cl2—O5'	1.434 (8)
C20—H20	0.9300	Cl2—O5	1.435 (9)
C22—H22A	0.9700	Cl2—O7	1.437 (8)
C22—H22B	0.9700	Cl2—O8'	1.439 (9)
C23—C28	1.37 (2)	Cl2—O6'	1.452 (9)
C23—C24	1.39 (2)	Cl2—O8	1.453 (9)
			
N2—Pt1—C16	82.8 (6)	C23—C28—H28	120.8
N2—Pt1—N3	178.1 (4)	C27—C28—H28	120.8
C16—Pt1—N3	98.7 (6)	C34—C29—C30	119.3 (14)
N2—Pt1—N1	79.5 (5)	C34—C29—P1	122.1 (12)
C16—Pt1—N1	162.3 (6)	C30—C29—P1	118.6 (13)
N3—Pt1—N1	99.0 (5)	C31—C30—C29	118 (2)
N6—Pt2—C50	81.7 (6)	C31—C30—H30	121.1
N6—Pt2—N5	77.4 (5)	C29—C30—H30	121.1
C50—Pt2—N5	158.8 (6)	C32—C31—C30	123 (2)
N6—Pt2—P1	173.4 (4)	C32—C31—H31	118.7
C50—Pt2—P1	95.0 (5)	C30—C31—H31	118.7
N5—Pt2—P1	106.2 (4)	C31—C32—C33	120.4 (18)
C22—P1—C29	102.7 (7)	C31—C32—H32	119.8
C22—P1—C23	104.9 (7)	C33—C32—H32	119.8
C29—P1—C23	103.7 (7)	C34—C33—C32	120 (2)
C22—P1—Pt2	114.5 (5)	C34—C33—H33	119.9
C29—P1—Pt2	115.5 (5)	C32—C33—H33	119.9
C23—P1—Pt2	114.2 (5)	C33—C34—C29	119.7 (19)
C1—N1—C5	118.6 (13)	C33—C34—H34	120.1
C1—N1—Pt1	130.0 (12)	C29—C34—H34	120.1
C5—N1—Pt1	111.3 (10)	N5—C35—C36	121 (2)
C6—N2—C10	120.0 (14)	N5—C35—H35	119.4
C6—N2—Pt1	121.8 (11)	C36—C35—H35	119.4
C10—N2—Pt1	117.9 (9)	C37—C36—C35	121 (2)
C21—N3—C17	117.7 (15)	C37—C36—H36	119.6
C21—N3—Pt1	122.7 (10)	C35—C36—H36	119.6
C17—N3—Pt1	119.3 (12)	C36—C37—C38	117.7 (17)
C21—N4—C22	123.9 (14)	C36—C37—H37	121.2
C21—N4—H4	118.1	C38—C37—H37	121.2
C22—N4—H4	118.1	C37—C38—C39	120 (2)
C35—N5—C39	120.2 (15)	C37—C38—H38	119.9
C35—N5—Pt2	128.9 (13)	C39—C38—H38	119.9
C39—N5—Pt2	110.8 (11)	N5—C39—C38	119.5 (16)
C40—N6—C44	121.2 (15)	N5—C39—C40	118.8 (15)
C40—N6—Pt2	120.5 (11)	C38—C39—C40	121.7 (17)
C44—N6—Pt2	117.4 (11)	N6—C40—C41	121.1 (18)
N1—C1—C2	122.9 (18)	N6—C40—C39	111.9 (15)
N1—C1—H1	118.6	C41—C40—C39	126.9 (19)
C2—C1—H1	118.6	C42—C41—C40	120 (2)
C3—C2—C1	119.2 (18)	C42—C41—H41	119.9
C3—C2—H2	120.4	C40—C41—H41	119.9
C1—C2—H2	120.4	C41—C42—C43	118.9 (19)
C2—C3—C4	120.3 (16)	C41—C42—H42	120.6
C2—C3—H3	119.9	C43—C42—H42	120.6
C4—C3—H3	119.9	C44—C43—C42	119.6 (18)
C5—C4—C3	119.6 (18)	C44—C43—H43	120.2
C5—C4—H4A	120.2	C42—C43—H43	120.2
C3—C4—H4A	120.2	N6—C44—C43	118.6 (17)
C4—C5—N1	119.2 (16)	N6—C44—C45	113.6 (15)
C4—C5—C6	124.8 (16)	C43—C44—C45	127.7 (16)
N1—C5—C6	115.9 (12)	C46—C45—C44	123.5 (17)
N2—C6—C7	121.5 (15)	C46—C45—C50	121.4 (17)
N2—C6—C5	111.3 (15)	C44—C45—C50	115.2 (13)
C7—C6—C5	127.0 (13)	C45—C46—C47	120.2 (18)
C6—C7—C8	119.5 (15)	C45—C46—H46	119.9
C6—C7—H7	120.2	C47—C46—H46	119.9
C8—C7—H7	120.2	C48—C47—C46	119.6 (17)
C9—C8—C7	118.6 (16)	C48—C47—H47	120.2
C9—C8—H8	120.7	C46—C47—H47	120.2
C7—C8—H8	120.7	C49—C48—C47	120 (2)
C8—C9—C10	121.8 (15)	C49—C48—H48	119.9
C8—C9—H9	119.1	C47—C48—H48	119.9
C10—C9—H9	119.1	C48—C49—C50	123.8 (18)
C9—C10—N2	117.9 (12)	C48—C49—H49	118.1
C9—C10—C11	131.1 (13)	C50—C49—H49	118.1
N2—C10—C11	110.3 (13)	C49—C50—C45	114.7 (13)
C16—C11—C12	120.6 (13)	C49—C50—Pt2	134.0 (12)
C16—C11—C10	117.9 (12)	C45—C50—Pt2	111.3 (12)
C12—C11—C10	121.3 (15)	O4—Cl1—O3	112.5 (8)
C13—C12—C11	118.4 (17)	O4—Cl1—O3'	134.0 (12)
C13—C12—H12	120.8	O4—Cl1—O2'	62.8 (14)
C11—C12—H12	120.8	O3—Cl1—O2'	112.1 (14)
C14—C13—C12	121.0 (16)	O3'—Cl1—O2'	111.2 (8)
C14—C13—H13	119.5	O4—Cl1—O1'	114.2 (13)
C12—C13—H13	119.5	O3—Cl1—O1'	127.1 (13)
C13—C14—C15	121.8 (15)	O3'—Cl1—O1'	110.3 (8)
C13—C14—H14	119.1	O2'—Cl1—O1'	110.3 (8)
C15—C14—H14	119.1	O4—Cl1—O1	110.9 (8)
C16—C15—C14	120.3 (17)	O3—Cl1—O1	110.4 (8)
C16—C15—H15	119.9	O3'—Cl1—O1	102.8 (13)
C14—C15—H15	119.9	O2'—Cl1—O1	135.7 (12)
C15—C16—C11	117.8 (14)	O4—Cl1—O4'	46.4 (13)
C15—C16—Pt1	131.4 (14)	O3—Cl1—O4'	86.9 (13)
C11—C16—Pt1	110.7 (9)	O3'—Cl1—O4'	108.4 (8)
C18—C17—N3	122.5 (17)	O2'—Cl1—O4'	108.0 (8)
C18—C17—H17	118.8	O1'—Cl1—O4'	108.5 (8)
N3—C17—H17	118.8	O1—Cl1—O4'	86.3 (12)
C17—C18—C19	120.9 (17)	O4—Cl1—O2	108.1 (8)
C17—C18—H18	119.5	O3—Cl1—O2	107.8 (8)
C19—C18—H18	119.5	O3'—Cl1—O2	90.2 (12)
C20—C19—C18	116.7 (16)	O2'—Cl1—O2	47.4 (13)
C20—C19—H19	121.6	O1'—Cl1—O2	79.8 (12)
C18—C19—H19	121.6	O1—Cl1—O2	107.0 (7)
C19—C20—C21	120.1 (15)	O4'—Cl1—O2	154.4 (13)
C19—C20—H20	120.0	O6—Cl2—O7'	111.9 (14)
C21—C20—H20	120.0	O6—Cl2—O5'	137.2 (13)
N3—C21—N4	116.8 (14)	O7'—Cl2—O5'	110.9 (8)
N3—C21—C20	122.0 (14)	O6—Cl2—O5	110.9 (8)
N4—C21—C20	121.2 (14)	O7'—Cl2—O5	129.3 (14)
N4—C22—P1	109.6 (11)	O6—Cl2—O7	110.4 (8)
N4—C22—H22A	109.8	O5'—Cl2—O7	107.4 (14)
P1—C22—H22A	109.8	O5—Cl2—O7	109.5 (8)
N4—C22—H22B	109.8	O6—Cl2—O8'	53.3 (14)
P1—C22—H22B	109.8	O7'—Cl2—O8'	110.0 (8)
H22A—C22—H22B	108.2	O5'—Cl2—O8'	109.9 (8)
C28—C23—C24	119.8 (14)	O5—Cl2—O8'	116.9 (15)
C28—C23—P1	119.0 (12)	O7—Cl2—O8'	133.6 (14)
C24—C23—P1	120.8 (13)	O6—Cl2—O6'	57.0 (13)
C25—C24—C23	119.6 (17)	O7'—Cl2—O6'	109.1 (8)
C25—C24—H24	120.2	O5'—Cl2—O6'	108.7 (8)
C23—C24—H24	120.2	O5—Cl2—O6'	73.9 (14)
C26—C25—C24	121.8 (18)	O7—Cl2—O6'	84.2 (13)
C26—C25—H25	119.1	O8'—Cl2—O6'	108.3 (8)
C24—C25—H25	119.1	O6—Cl2—O8	108.4 (8)
C25—C26—C27	117.7 (16)	O7'—Cl2—O8	82.0 (12)
C25—C26—H26	121.2	O5'—Cl2—O8	76.8 (14)
C27—C26—H26	121.2	O5—Cl2—O8	108.6 (8)
C26—C27—C28	122.5 (18)	O7—Cl2—O8	109.0 (8)
C26—C27—H27	118.8	O8'—Cl2—O8	55.9 (14)
C28—C27—H27	118.8	O6'—Cl2—O8	163.8 (14)
C23—C28—C27	118.5 (16)		
			
C50—Pt2—P1—C22	−59.8 (6)	Pt1—N3—C21—C20	−175.0 (9)
N5—Pt2—P1—C22	118.4 (6)	C22—N4—C21—N3	−161.1 (12)
C50—Pt2—P1—C29	59.2 (6)	C22—N4—C21—C20	20.9 (19)
N5—Pt2—P1—C29	−122.6 (6)	C19—C20—C21—N3	3(2)
C50—Pt2—P1—C23	179.3 (7)	C19—C20—C21—N4	−179.1 (13)
N5—Pt2—P1—C23	−2.5 (7)	C21—N4—C22—P1	−178.1 (10)
N2—Pt1—N1—C1	174.6 (16)	C29—P1—C22—N4	−148.3 (9)
C16—Pt1—N1—C1	173.0 (18)	C23—P1—C22—N4	103.6 (10)
N3—Pt1—N1—C1	−6.6 (16)	Pt2—P1—C22—N4	−22.4 (10)
N2—Pt1—N1—C5	−2.8 (10)	C22—P1—C23—C28	155.5 (14)
C16—Pt1—N1—C5	−4(2)	C29—P1—C23—C28	48.1 (16)
N3—Pt1—N1—C5	176.0 (10)	Pt2—P1—C23—C28	−78.4 (15)
C16—Pt1—N2—C6	−176.1 (12)	C22—P1—C23—C24	−31.1 (16)
N1—Pt1—N2—C6	4.4 (11)	C29—P1—C23—C24	−138.5 (14)
C16—Pt1—N2—C10	−2.9 (11)	Pt2—P1—C23—C24	95.0 (14)
N1—Pt1—N2—C10	177.6 (11)	C28—C23—C24—C25	5(3)
C16—Pt1—N3—C21	−72.3 (11)	P1—C23—C24—C25	−168.3 (16)
N1—Pt1—N3—C21	107.6 (11)	C23—C24—C25—C26	−3(3)
C16—Pt1—N3—C17	114.7 (11)	C24—C25—C26—C27	1(3)
N1—Pt1—N3—C17	−65.4 (11)	C25—C26—C27—C28	0(3)
N6—Pt2—N5—C35	177.4 (17)	C24—C23—C28—C27	−4(3)
C50—Pt2—N5—C35	166.7 (16)	P1—C23—C28—C27	169.1 (16)
P1—Pt2—N5—C35	−8.3 (17)	C26—C27—C28—C23	2(3)
N6—Pt2—N5—C39	−6.1 (11)	C22—P1—C29—C34	−45.2 (13)
C50—Pt2—N5—C39	−17 (2)	C23—P1—C29—C34	63.8 (13)
P1—Pt2—N5—C39	168.1 (10)	Pt2—P1—C29—C34	−170.5 (10)
C50—Pt2—N6—C40	−176.6 (13)	C22—P1—C29—C30	134.6 (11)
N5—Pt2—N6—C40	7.4 (12)	C23—P1—C29—C30	−116.4 (12)
C50—Pt2—N6—C44	−7.6 (10)	Pt2—P1—C29—C30	9.3 (12)
N5—Pt2—N6—C44	176.3 (11)	C34—C29—C30—C31	−1(2)
C5—N1—C1—C2	−3(3)	P1—C29—C30—C31	179.5 (12)
Pt1—N1—C1—C2	−180.0 (13)	C29—C30—C31—C32	−1(3)
N1—C1—C2—C3	1(3)	C30—C31—C32—C33	3(3)
C1—C2—C3—C4	2(3)	C31—C32—C33—C34	−3(3)
C2—C3—C4—C5	−3(3)	C32—C33—C34—C29	1(2)
C3—C4—C5—N1	1(2)	C30—C29—C34—C33	1(2)
C3—C4—C5—C6	179.2 (16)	P1—C29—C34—C33	−179.5 (12)
C1—N1—C5—C4	2(2)	C39—N5—C35—C36	2(3)
Pt1—N1—C5—C4	179.5 (12)	Pt2—N5—C35—C36	178.0 (16)
C1—N1—C5—C6	−176.5 (14)	N5—C35—C36—C37	−5(4)
Pt1—N1—C5—C6	1.3 (16)	C35—C36—C37—C38	7(4)
C10—N2—C6—C7	7(2)	C36—C37—C38—C39	−6(3)
Pt1—N2—C6—C7	−179.7 (12)	C35—N5—C39—C38	−2(3)
C10—N2—C6—C5	−177.9 (12)	Pt2—N5—C39—C38	−178.4 (14)
Pt1—N2—C6—C5	−4.7 (17)	C35—N5—C39—C40	−178.5 (17)
C4—C5—C6—N2	−176.2 (14)	Pt2—N5—C39—C40	4.7 (19)
N1—C5—C6—N2	1.9 (19)	C37—C38—C39—N5	4(3)
C4—C5—C6—C7	−2(3)	C37—C38—C39—C40	−179.3 (18)
N1—C5—C6—C7	176.5 (15)	C44—N6—C40—C41	8(3)
N2—C6—C7—C8	−3(2)	Pt2—N6—C40—C41	176.8 (13)
C5—C6—C7—C8	−177.0 (16)	C44—N6—C40—C39	−175.5 (13)
C6—C7—C8—C9	1(3)	Pt2—N6—C40—C39	−6.9 (19)
C7—C8—C9—C10	−3(3)	N5—C39—C40—N6	1(2)
C8—C9—C10—N2	7(2)	C38—C39—C40—N6	−176.0 (16)
C8—C9—C10—C11	177.0 (17)	N5—C39—C40—C41	176.8 (17)
C6—N2—C10—C9	−9(2)	C38—C39—C40—C41	0(3)
Pt1—N2—C10—C9	177.6 (11)	N6—C40—C41—C42	−6(3)
C6—N2—C10—C11	179.0 (12)	C39—C40—C41—C42	178.6 (18)
Pt1—N2—C10—C11	5.6 (16)	C40—C41—C42—C43	0(3)
C9—C10—C11—C16	−176.9 (16)	C41—C42—C43—C44	3(3)
N2—C10—C11—C16	−6.3 (19)	C40—N6—C44—C43	−5(2)
C9—C10—C11—C12	8(3)	Pt2—N6—C44—C43	−173.6 (12)
N2—C10—C11—C12	178.9 (13)	C40—N6—C44—C45	179.0 (14)
C16—C11—C12—C13	2(2)	Pt2—N6—C44—C45	10.1 (17)
C10—C11—C12—C13	176.4 (16)	C42—C43—C44—N6	−1(3)
C11—C12—C13—C14	−2(3)	C42—C43—C44—C45	174.6 (17)
C12—C13—C14—C15	2(3)	N6—C44—C45—C46	172.9 (14)
C13—C14—C15—C16	−3(3)	C43—C44—C45—C46	−3(3)
C14—C15—C16—C11	3(2)	N6—C44—C45—C50	−7(2)
C14—C15—C16—Pt1	−178.8 (13)	C43—C44—C45—C50	176.9 (15)
C12—C11—C16—C15	−3(2)	C44—C45—C46—C47	−176.8 (16)
C10—C11—C16—C15	−177.5 (14)	C50—C45—C46—C47	3(3)
C12—C11—C16—Pt1	179.0 (11)	C45—C46—C47—C48	−5(3)
C10—C11—C16—Pt1	4.1 (17)	C46—C47—C48—C49	2(3)
N2—Pt1—C16—C15	−178.8 (16)	C47—C48—C49—C50	2(3)
N3—Pt1—C16—C15	2.4 (17)	C48—C49—C50—C45	−4(2)
N1—Pt1—C16—C15	−177.2 (15)	C48—C49—C50—Pt2	176.0 (13)
N2—Pt1—C16—C11	−0.7 (11)	C46—C45—C50—C49	1(2)
N3—Pt1—C16—C11	−179.5 (10)	C44—C45—C50—C49	−179.1 (14)
N1—Pt1—C16—C11	1(3)	C46—C45—C50—Pt2	−179.0 (12)
C21—N3—C17—C18	2(2)	C44—C45—C50—Pt2	1.2 (16)
Pt1—N3—C17—C18	175.7 (12)	N6—Pt2—C50—C49	−176.6 (17)
N3—C17—C18—C19	−4(2)	N5—Pt2—C50—C49	−165.9 (15)
C17—C18—C19—C20	4(2)	P1—Pt2—C50—C49	9.2 (16)
C18—C19—C20—C21	−4(2)	N6—Pt2—C50—C45	3.1 (10)
C17—N3—C21—N4	−179.9 (12)	N5—Pt2—C50—C45	14 (2)
Pt1—N3—C21—N4	6.9 (16)	P1—Pt2—C50—C45	−171.1 (9)
C17—N3—C21—C20	−1.9 (19)		

## References

[bb1] Braunstein, P., Charles, C., Braunstein, P., Charles, C., Kickelbick, G. & Schubert, U. (1997). *Chem. Commun.* pp. 1911–1912.

[bb2] Bruker (2007). *SMART* and *SAINT* Bruker AXS Inc., Madison, Wisconsin, USA.

[bb3] Catalano, V. J., Bennett, B. L., Muratidis, S. & Noll, B. C. (2001). *J. Am. Chem. Soc.* **123**, 173–174. 10.1021/ja0055054 11273614

[bb4] Durran, S. E., Smith, M. B., Slawin, A. M. Z. & Steed, J. W. (2000). *J. Chem. Soc. Dalton Trans.* pp. 2771–2778.

[bb5] Field, J. S., Haines, R. J. & Parry, C. J. (1997). *J. Chem. Soc. Dalton Trans.* pp. 2843–2848.

[bb6] Kuang, S. M., Zhang, Z. Z., Wang, Q. G. & Mak, T. C. W. (1998). *Inorg. Chem.* **37**, 6090–6092. 10.1021/ic9800482 11670748

[bb7] Li, S. L., Mak, T. C. W. & Zhang, Z. Z. (1996). J. Chem. Soc. Dalton Trans. pp. 3475–3483.

[bb8] Newkome, G. R. (1993). *Chem. Rev.* **93**, 2067–2089.

[bb9] Sheldrick, G. M. (1996). *SADABS* University of Göttingen, Germany.

[bb10] Sheldrick, G. M. (2008). *Acta Cryst.* A**64**, 112–122. 10.1107/S0108767307043930 18156677

